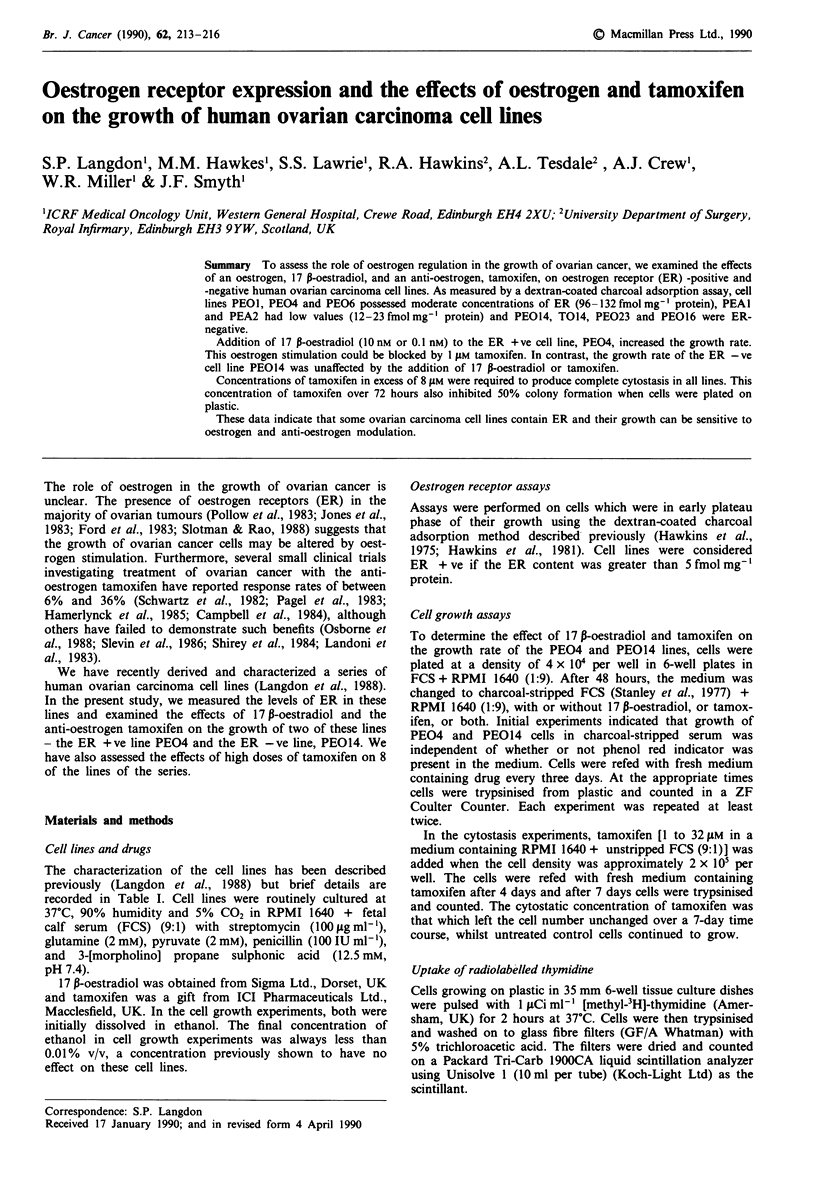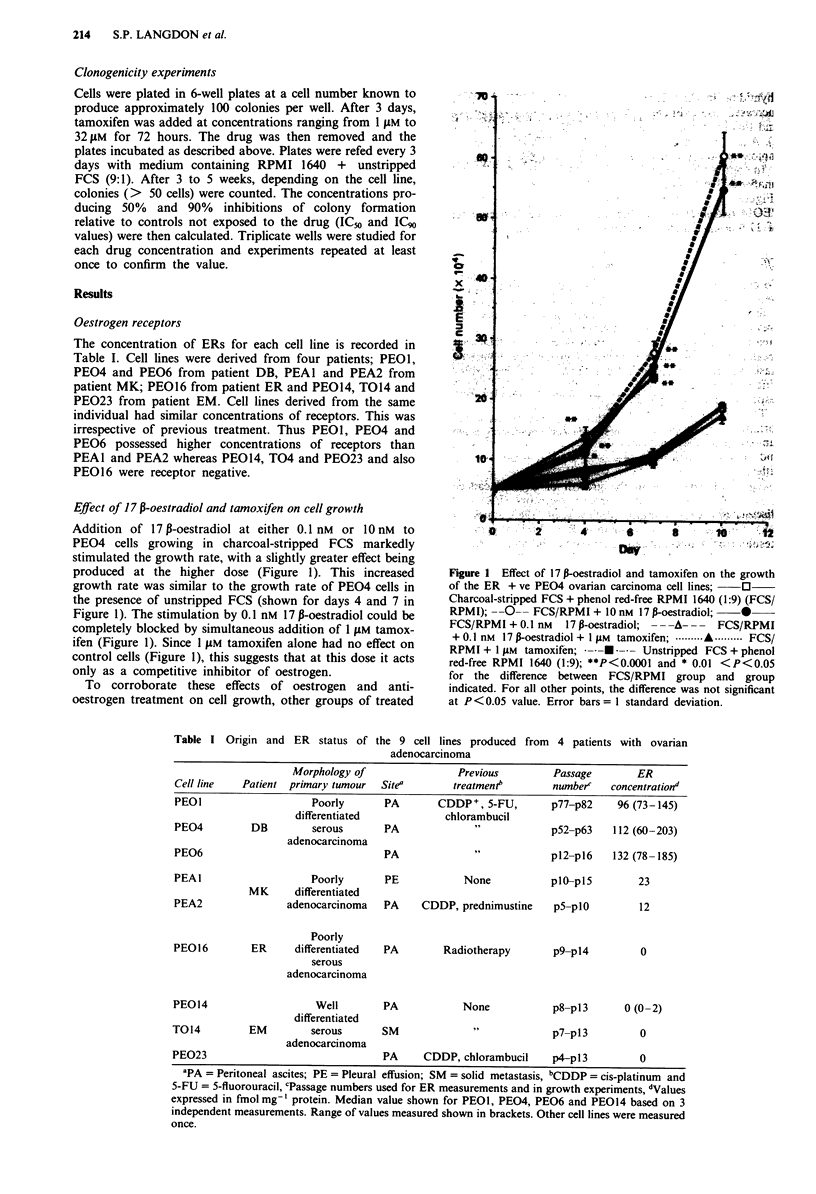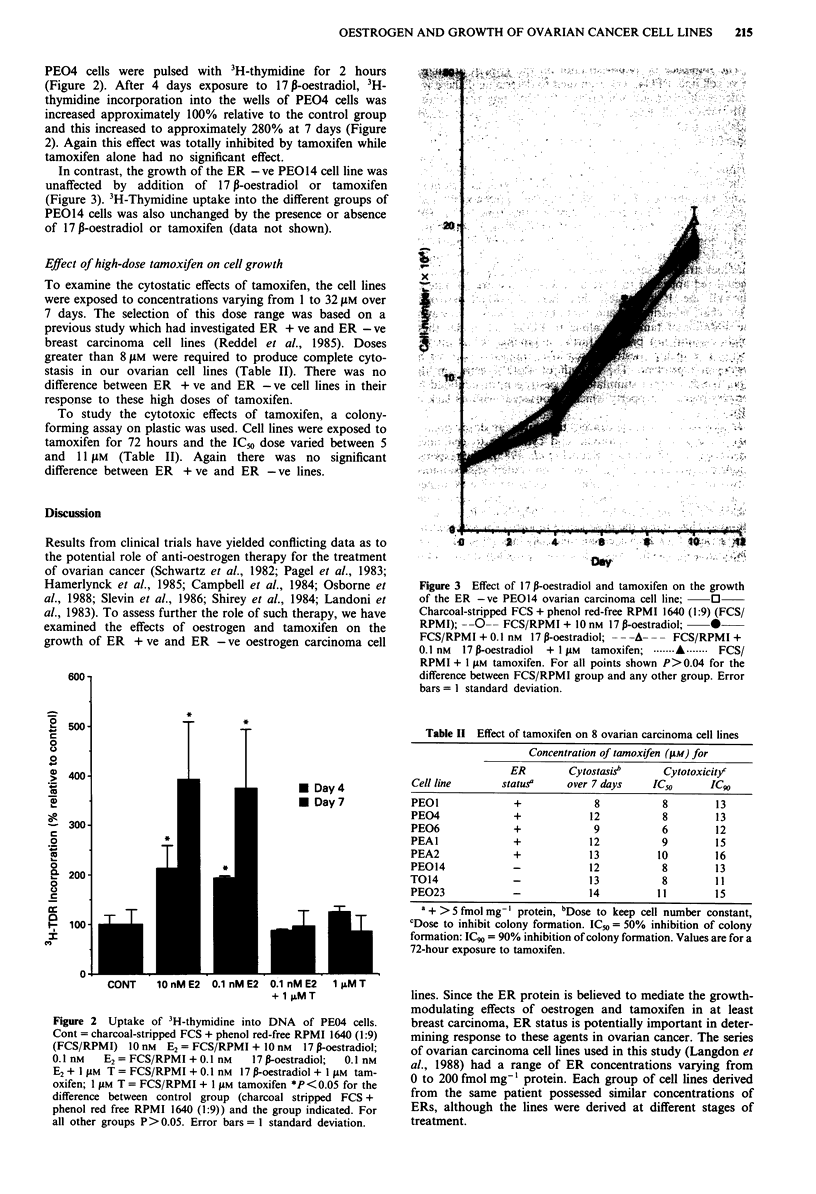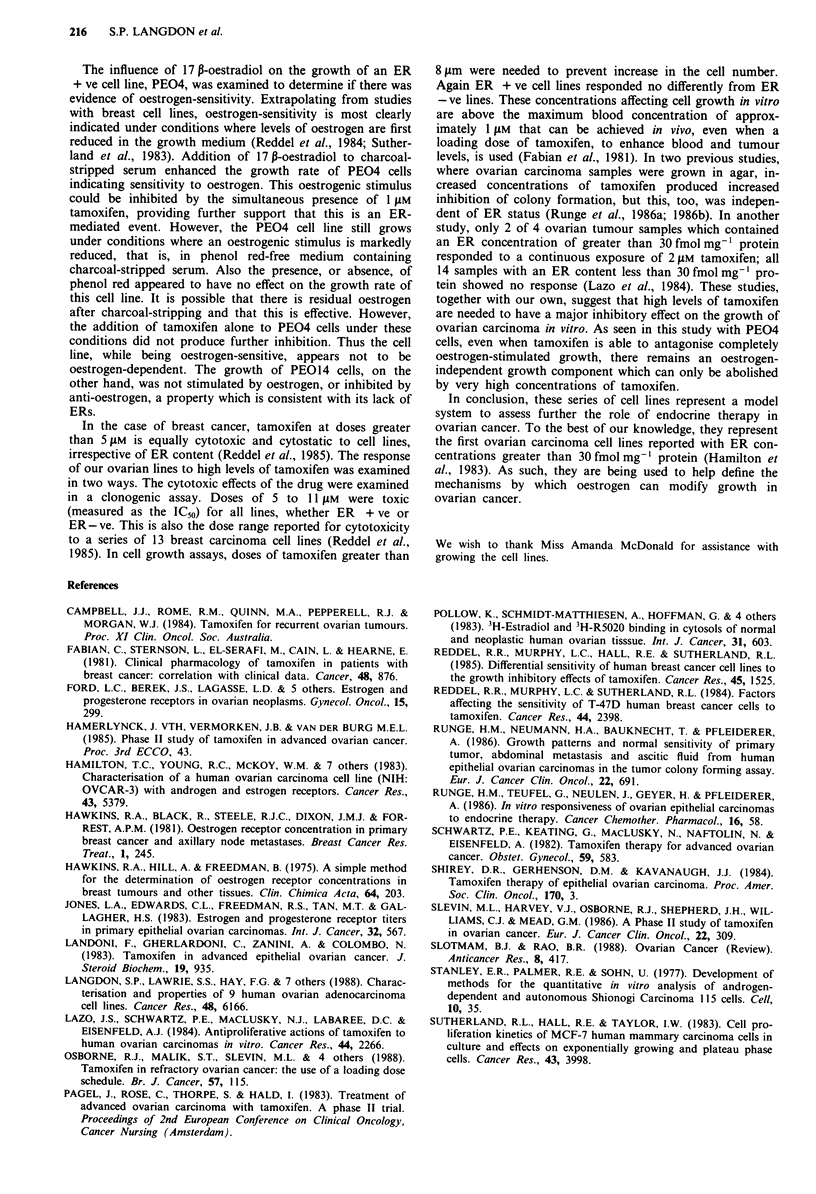# Oestrogen receptor expression and the effects of oestrogen and tamoxifen on the growth of human ovarian carcinoma cell lines.

**DOI:** 10.1038/bjc.1990.263

**Published:** 1990-08

**Authors:** S. P. Langdon, M. M. Hawkes, S. S. Lawrie, R. A. Hawkins, A. L. Tesdale, A. J. Crew, W. R. Miller, J. F. Smyth

**Affiliations:** ICRF Medical Oncology Unit, Western General Hospital, Edinburgh, Scotland, UK.

## Abstract

To assess the role of oestrogen regulation in the growth of ovarian cancer, we examined the effects of an oestrogen, 17 beta-oestradiol, and an anti-oestrogen, tamoxifen, on oestrogen receptor (ER) -positive and -negative human ovarian carcinoma cell lines. As measured by a dextran-coated charcoal adsorption assay, cell lines PEO1, PEO4 and PEO6 possessed moderate concentrations of ER (96-132 fmol mg-1 protein), PEA1 and PEA2 had low values (12-23 fmol mg-1 protein) and PEO14, TO14, PEO23 and PEO16 were ER-negative. Addition of 17 beta-oestradiol (10 nM or 0.1 nM) to the ER +ve cell line, PEO4, increased the growth rate. This oestrogen stimulation could be blocked by 1 microM tamoxifen. In contrast, the growth rate of the ER -ve cell line PEO14 was unaffected by the addition of 17 beta-oestradiol or tamoxifen. Concentrations of tamoxifen in excess of 8 microM were required to produce complete cytostasis in all lines. This concentration of tamoxifen over 72 hours also inhibited 50% colony formation when cells were plated on plastic. These data indicate that some ovarian carcinoma cell lines contain ER and their growth can be sensitive to oestrogen and anti-oestrogen modulation.


Br. J. Cancer (1990), 62, 213-216
© Macmillan Press Ltd., 1990

### Oestrogen receptor expression and the effects of oestrogen and tamoxifen on the growth of human ovarian carcinoma cell lines

S.P. Langdon[1], M.M. Hawkes[1], S.S. Lawrie[1], R.A. Hawkins[2], A.L. Tesdale[2], A.J. Crew[1], W.R. Miller[1] & J.F. Smyth[1]

[1]*ICRF Medical Oncology Unit, Western General Hospital, Crewe Road, Edinburgh EH4 2XU;* [2]*University Department of Surgery, Royal Infirmary, Edinburgh EH3 9YW, Scotland, UK*

**Summary** To assess the role of oestrogen regulation in the growth of ovarian cancer, we examined the effects of an oestrogen, 17 β-oestradiol, and an anti-oestrogen, tamoxifen, on oestrogen receptor (ER) -positive and -negative human ovarian carcinoma cell lines. As measured by a dextran-coated charcoal adsorption assay, cell lines PEO1, PEO4 and PEO6 possessed moderate concentrations of ER (96-132 fmol $mg^{-1}$ protein), PEA1 and PEA2 had low values (12-23 fmol $mg^{-1}$ protein) and PEO14, TO14, PEO23 and PEO16 were ER-negative.

Addition of 17 β-oestradiol (10 nM or 0.1 nM) to the ER +ve cell line, PEO4, increased the growth rate. This oestrogen stimulation could be blocked by 1 μM tamoxifen. In contrast, the growth rate of the ER −ve cell line PEO14 was unaffected by the addition of 17 β-oestradiol or tamoxifen.

Concentrations of tamoxifen in excess of 8 μM were required to produce complete cytostasis in all lines. This concentration of tamoxifen over 72 hours also inhibited 50% colony formation when cells were plated on plastic.

These data indicate that some ovarian carcinoma cell lines contain ER and their growth can be sensitive to oestrogen and anti-oestrogen modulation.

The role of oestrogen in the growth of ovarian cancer is unclear. The presence of oestrogen receptors (ER) in the majority of ovarian tumours (Pollow *et al.*, 1983; Jones *et al.*, 1983; Ford *et al.*, 1983; Slotman & Rao, 1988) suggests that the growth of ovarian cancer cells may be altered by oestrogen stimulation. Furthermore, several small clinical trials investigating treatment of ovarian cancer with the anti-oestrogen tamoxifen have reported response rates of between 6% and 36% (Schwartz *et al.*, 1982; Pagel *et al.*, 1983; Hamerlynck *et al.*, 1985; Campbell *et al.*, 1984), although others have failed to demonstrate such benefits (Osborne *et al.*, 1988; Slevin *et al.*, 1986; Shirey *et al.*, 1984; Landoni *et al.*, 1983).

We have recently derived and characterized a series of human ovarian carcinoma cell lines (Langdon *et al.*, 1988). In the present study, we measured the levels of ER in these lines and examined the effects of 17 β-oestradiol and the anti-oestrogen tamoxifen on the growth of two of these lines - the ER +ve line PEO4 and the ER −ve line, PEO14. We have also assessed the effects of high doses of tamoxifen on 8 of the lines of the series.

### Materials and methods

### *Cell lines and drugs*

The characterization of the cell lines has been described previously (Langdon *et al.*, 1988) but brief details are recorded in Table I. Cell lines were routinely cultured at 37°C, 90% humidity and 5% $CO_2$ in RPMI 1640 + fetal calf serum (FCS) (9:1) with streptomycin (100 μg $ml^{-1}$), glutamine (2 mM), pyruvate (2 mM), penicillin (100 IU $ml^{-1}$), and 3-[morpholino] propane sulphonic acid (12.5 mM, pH 7.4).

17 β-oestradiol was obtained from Sigma Ltd., Dorset, UK and tamoxifen was a gift from ICI Pharmaceuticals Ltd., Macclesfield, UK. In the cell growth experiments, both were initially dissolved in ethanol. The final concentration of ethanol in cell growth experiments was always less than 0.01% v/v, a concentration previously shown to have no effect on these cell lines.

### *Oestrogen receptor assays*

Assays were performed on cells which were in early plateau phase of their growth using the dextran-coated charcoal adsorption method described previously (Hawkins *et al.*, 1975; Hawkins *et al.*, 1981). Cell lines were considered ER +ve if the ER content was greater than 5 fmol $mg^{-1}$ protein.

### *Cell growth assays*

To determine the effect of 17 β-oestradiol and tamoxifen on the growth rate of the PEO4 and PEO14 lines, cells were plated at a density of $4 \times 10^4$ per well in 6-well plates in FCS + RPMI 1640 (1:9). After 48 hours, the medium was changed to charcoal-stripped FCS (Stanley *et al.*, 1977) + RPMI 1640 (1:9), with or without 17 β-oestradiol, or tamoxifen, or both. Initial experiments indicated that growth of PEO4 and PEO14 cells in charcoal-stripped serum was independent of whether or not phenol red indicator was present in the medium. Cells were refed with fresh medium containing drug every three days. At the appropriate times cells were trypsinised from plastic and counted in a ZF Coulter Counter. Each experiment was repeated at least twice.

In the cytostasis experiments, tamoxifen [1 to 32 μM in a medium containing RPMI 1640 + unstripped FCS (9:1)] was added when the cell density was approximately $2 \times 10^5$ per well. The cells were refed with fresh medium containing tamoxifen after 4 days and after 7 days cells were trypsinised and counted. The cytostatic concentration of tamoxifen was that which left the cell number unchanged over a 7-day time course, whilst untreated control cells continued to grow.

### *Uptake of radiolabelled thymidine*

Cells growing on plastic in 35 mm 6-well tissue culture dishes were pulsed with 1 μCi $ml^{-1}$ [methyl-$^3$H]-thymidine (Amersham, UK) for 2 hours at 37°C. Cells were then trypsinised and washed on to glass fibre filters (GF/A Whatman) with 5% trichloroacetic acid. The filters were dried and counted on a Packard Tri-Carb 1900CA liquid scintillation analyzer using Unisolve 1 (10 ml per tube) (Koch-Light Ltd) as the scintillant.

Correspondence: S.P. Langdon
Received 17 January 1990; and in revised form 4 April 1990

*Clonogenicity experiments*

Cells were plated in 6-well plates at a cell number known to produce approximately 100 colonies per well. After 3 days, tamoxifen was added at concentrations ranging from 1 μM to 32 μM for 72 hours. The drug was then removed and the plates incubated as described above. Plates were refed every 3 days with medium containing RPMI 1640 + unstripped FCS (9:1). After 3 to 5 weeks, depending on the cell line, colonies (> 50 cells) were counted. The concentrations producing 50% and 90% inhibitions of colony formation relative to controls not exposed to the drug ($IC_{50}$ and $IC_{90}$ values) were then calculated. Triplicate wells were studied for each drug concentration and experiments repeated at least once to confirm the value.

### Results

*Oestrogen receptors*

The concentration of ERs for each cell line is recorded in Table I. Cell lines were derived from four patients; PEO1, PEO4 and PEO6 from patient DB, PEA1 and PEA2 from patient MK; PEO16 from patient ER and PEO14, TO14 and PEO23 from patient EM. Cell lines derived from the same individual had similar concentrations of receptors. This was irrespective of previous treatment. Thus PEO1, PEO4 and PEO6 possessed higher concentrations of receptors than PEA1 and PEA2 whereas PEO14, TO4 and PEO23 and also PEO16 were receptor negative.

*Effect of 17 β-oestradiol and tamoxifen on cell growth*

Addition of 17β-oestradiol at either 0.1 nM or 10 nM to PEO4 cells growing in charcoal-stripped FCS markedly stimulated the growth rate, with a slightly greater effect being produced at the higher dose (Figure 1). This increased growth rate was similar to the growth rate of PEO4 cells in the presence of unstripped FCS (shown for days 4 and 7 in Figure 1). The stimulation by 0.1 nM 17 β-oestradiol could be completely blocked by simultaneous addition of 1 μM tamoxifen (Figure 1). Since 1 μM tamoxifen alone had no effect on control cells (Figure 1), this suggests that at this dose it acts only as a competitive inhibitor of oestrogen.

To corroborate these effects of oestrogen and anti-oestrogen treatment on cell growth, other groups of treated

**Figure 1** Effect of 17 β-oestradiol and tamoxifen on the growth of the ER + ve PEO4 ovarian carcinoma cell lines; ——□—— Charcoal-stripped FCS + phenol red-free RPMI 1640 (1:9) (FCS/RPMI); --○-- FCS/RPMI + 10 nM 17 β-oestradiol; ——●—— FCS/RPMI + 0.1 nM 17 β-oestradiol; ---△--- FCS/RPMI + 0.1 nM 17 β-oestradiol + 1 μM tamoxifen; ·········▲········· FCS/RPMI + 1 μM tamoxifen; -·-■-·- Unstripped FCS + phenol red-free RPMI 1640 (1:9); $^{**}P<0.0001$ and * $0.01<P<0.05$ for the difference between FCS/RPMI group and group indicated. For all other points, the difference was not significant at $P<0.05$ value. Error bars = 1 standard deviation.

**Table I** Origin and ER status of the 9 cell lines produced from 4 patients with ovarian adenocarcinoma

| *Cell line* | *Patient* | *Morphology of primary tumour* | *Site*[a] | *Previous treatment*[b] | *Passage number*[c] | *ER concentration*[d] |
|---|---|---|---|---|---|---|
| PEO1 | | Poorly differentiated | PA | CDDP+, 5-FU, chlorambucil | p77–p82 | 96 (73–145) |
| PEO4 | DB | serous adenocarcinoma | PA | " | p52–p63 | 112 (60–203) |
| PEO6 | | | PA | " | p12–p16 | 132 (78–185) |
| PEA1 | | Poorly | PE | None | p10–p15 | 23 |
| | MK | differentiated | | | | |
| PEA2 | | adenocarcinoma | PA | CDDP, prednimustine | p5–p10 | 12 |
| PEO16 | ER | Poorly differentiated serous adenocarcinoma | PA | Radiotherapy | p9–p14 | 0 |
| PEO14 | | Well differentiated | PA | None | p8–p13 | 0 (0–2) |
| TO14 | EM | serous adenocarcinoma | SM | " | p7–p13 | 0 |
| PEO23 | | | PA | CDDP, chlorambucil | p4–p13 | 0 |

[a]PA = Peritoneal ascites; PE = Pleural effusion; SM = solid metastasis, [b]CDDP = cis-platinum and 5-FU = 5-fluorouracil, [c]Passage numbers used for ER measurements and in growth experiments, [d]Values expressed in fmol $mg^{-1}$ protein. Median value shown for PEO1, PEO4, PEO6 and PEO14 based on 3 independent measurements. Range of values measured shown in brackets. Other cell lines were measured once.

PEO4 cells were pulsed with $^3$H-thymidine for 2 hours (Figure 2). After 4 days exposure to 17 $\beta$-oestradiol, $^3$H-thymidine incorporation into the wells of PEO4 cells was increased approximately 100% relative to the control group and this increased to approximately 280% at 7 days (Figure 2). Again this effect was totally inhibited by tamoxifen while tamoxifen alone had no significant effect.

In contrast, the growth of the ER −ve PEO14 cell line was unaffected by addition of 17 $\beta$-oestradiol or tamoxifen (Figure 3). $^3$H-Thymidine uptake into the different groups of PEO14 cells was also unchanged by the presence or absence of 17 $\beta$-oestradiol or tamoxifen (data not shown).

*Effect of high-dose tamoxifen on cell growth*

To examine the cytostatic effects of tamoxifen, the cell lines were exposed to concentrations varying from 1 to 32 $\mu$M over 7 days. The selection of this dose range was based on a previous study which had investigated ER + ve and ER − ve breast carcinoma cell lines (Reddel *et al.*, 1985). Doses greater than 8 $\mu$M were required to produce complete cytostasis in our ovarian cell lines (Table II). There was no difference between ER + ve and ER − ve cell lines in their response to these high doses of tamoxifen.

To study the cytotoxic effects of tamoxifen, a colony-forming assay on plastic was used. Cell lines were exposed to tamoxifen for 72 hours and the $IC_{50}$ dose varied between 5 and 11 $\mu$M (Table II). Again there was no significant difference between ER + ve and ER − ve lines.

### Discussion

Results from clinical trials have yielded conflicting data as to the potential role of anti-oestrogen therapy for the treatment of ovarian cancer (Schwartz *et al.*, 1982; Pagel *et al.*, 1983; Hamerlynck *et al.*, 1985; Campbell *et al.*, 1984; Osborne *et al.*, 1988; Slevin *et al.*, 1986; Shirey *et al.*, 1984; Landoni *et al.*, 1983). To assess further the role of such therapy, we have examined the effects of oestrogen and tamoxifen on the growth of ER + ve and ER − ve oestrogen carcinoma cell lines. Since the ER protein is believed to mediate the growth-modulating effects of oestrogen and tamoxifen in at least breast carcinoma, ER status is potentially important in determining response to these agents in ovarian cancer. The series of ovarian carcinoma cell lines used in this study (Langdon *et al.*, 1988) had a range of ER concentrations varying from 0 to 200 fmol mg$^{-1}$ protein. Each group of cell lines derived from the same patient possessed similar concentrations of ERs, although the lines were derived at different stages of treatment.

**Figure 2** Uptake of $^3$H-thymidine into DNA of PE04 cells. Cont = charcoal-stripped FCS + phenol red-free RPMI 1640 (1:9) (FCS/RPMI) 10 nM $E_2$ = FCS/RPMI + 10 nM 17 $\beta$-oestradiol; 0.1 nM $E_2$ = FCS/RPMI + 0.1 nM 17 $\beta$-oestradiol; 0.1 nM $E_2$ + 1 $\mu$M T = FCS/RPMI + 0.1 nM 17 $\beta$-oestradiol + 1 $\mu$M tamoxifen; 1 $\mu$M T = FCS/RPMI + 1 $\mu$M tamoxifen *$P < 0.05$ for the difference between control group (charcoal stripped FCS + phenol red free RPMI 1640 (1:9)) and the group indicated. For all other groups $P > 0.05$. Error bars = 1 standard deviation.

**Figure 3** Effect of 17 $\beta$-oestradiol and tamoxifen on the growth of the ER − ve PEO14 ovarian carcinoma cell line; ——□—— Charcoal-stripped FCS + phenol red-free RPMI 1640 (1:9) (FCS/RPMI); --○-- FCS/RPMI + 10 nM 17 $\beta$-oestradiol; ——●—— FCS/RPMI + 0.1 nM 17 $\beta$-oestradiol; ---△--- FCS/RPMI + 0.1 nM 17 $\beta$-oestradiol + 1 $\mu$M tamoxifen; ·······▲······· FCS/RPMI + 1 $\mu$M tamoxifen. For all points shown $P > 0.04$ for the difference between FCS/RPMI group and any other group. Error bars = 1 standard deviation.

**Table II** Effect of tamoxifen on 8 ovarian carcinoma cell lines

| | *Concentration of tamoxifen ($\mu$M) for* | | | |
|---|---|---|---|---|
| *Cell line* | *ER status*[a] | *Cytostasis*[b] *over 7 days* | *Cytotoxicity*[c] $IC_{50}$ | $IC_{90}$ |
| PEO1 | + | 8 | 8 | 13 |
| PEO4 | + | 12 | 8 | 13 |
| PEO6 | + | 9 | 6 | 12 |
| PEA1 | + | 12 | 9 | 15 |
| PEA2 | + | 13 | 10 | 16 |
| PEO14 | − | 12 | 8 | 13 |
| TO14 | − | 13 | 8 | 11 |
| PEO23 | − | 14 | 11 | 15 |

[a] + > 5 fmol mg$^{-1}$ protein, [b]Dose to keep cell number constant, [c]Dose to inhibit colony formation. $IC_{50}$ = 50% inhibition of colony formation: $IC_{90}$ = 90% inhibition of colony formation. Values are for a 72-hour exposure to tamoxifen.

The influence of 17 β-oestradiol on the growth of an ER + ve cell line, PEO4, was examined to determine if there was evidence of oestrogen-sensitivity. Extrapolating from studies with breast cell lines, oestrogen-sensitivity is most clearly indicated under conditions where levels of oestrogen are first reduced in the growth medium (Reddel et al., 1984; Sutherland et al., 1983). Addition of 17 β-oestradiol to charcoal-stripped serum enhanced the growth rate of PEO4 cells indicating sensitivity to oestrogen. This oestrogenic stimulus could be inhibited by the simultaneous presence of 1 μM tamoxifen, providing further support that this is an ER-mediated event. However, the PEO4 cell line still grows under conditions where an oestrogenic stimulus is markedly reduced, that is, in phenol red-free medium containing charcoal-stripped serum. Also the presence, or absence, of phenol red appeared to have no effect on the growth rate of this cell line. It is possible that there is residual oestrogen after charcoal-stripping and that this is effective. However, the addition of tamoxifen alone to PEO4 cells under these conditions did not produce further inhibition. Thus the cell line, while being oestrogen-sensitive, appears not to be oestrogen-dependent. The growth of PEO14 cells, on the other hand, was not stimulated by oestrogen, or inhibited by anti-oestrogen, a property which is consistent with its lack of ERs.

In the case of breast cancer, tamoxifen at doses greater than 5 μM is equally cytotoxic and cytostatic to cell lines, irrespective of ER content (Reddel et al., 1985). The response of our ovarian lines to high levels of tamoxifen was examined in two ways. The cytotoxic effects of the drug were examined in a clonogenic assay. Doses of 5 to 11 μM were toxic (measured as the $IC_{50}$) for all lines, whether ER + ve or ER − ve. This is also the dose range reported for cytotoxicity to a series of 13 breast carcinoma cell lines (Reddel et al., 1985). In cell growth assays, doses of tamoxifen greater than 8 μm were needed to prevent increase in the cell number. Again ER + ve cell lines responded no differently from ER − ve lines. These concentrations affecting cell growth *in vitro* are above the maximum blood concentration of approximately 1 μM that can be achieved *in vivo*, even when a loading dose of tamoxifen, to enhance blood and tumour levels, is used (Fabian et al., 1981). In two previous studies, where ovarian carcinoma samples were grown in agar, increased concentrations of tamoxifen produced increased inhibition of colony formation, but this, too, was independent of ER status (Runge et al., 1986a; 1986b). In another study, only 2 of 4 ovarian tumour samples which contained an ER concentration of greater than 30 fmol mg$^{-1}$ protein responded to a continuous exposure of 2 μM tamoxifen; all 14 samples with an ER content less than 30 fmol mg$^{-1}$ protein showed no response (Lazo et al., 1984). These studies, together with our own, suggest that high levels of tamoxifen are needed to have a major inhibitory effect on the growth of ovarian carcinoma *in vitro*. As seen in this study with PEO4 cells, even when tamoxifen is able to antagonise completely oestrogen-stimulated growth, there remains an oestrogen-independent growth component which can only be abolished by very high concentrations of tamoxifen.

In conclusion, these series of cell lines represent a model system to assess further the role of endocrine therapy in ovarian cancer. To the best of our knowledge, they represent the first ovarian carcinoma cell lines reported with ER concentrations greater than 30 fmol mg$^{-1}$ protein (Hamilton et al., 1983). As such, they are being used to help define the mechanisms by which oestrogen can modify growth in ovarian cancer.

We wish to thank Miss Amanda McDonald for assistance with growing the cell lines.

### References

CAMPBELL, J.J., ROME, R.M., QUINN, M.A., PEPPERELL, R.J. & MORGAN, W.J. (1984). Tamoxifen for recurrent ovarian tumours. *Proc. XI Clin. Oncol. Soc. Australia.*

FABIAN, C., STERNSON, L., EL-SERAFI, M., CAIN, L. & HEARNE, E. (1981). Clinical pharmacology of tamoxifen in patients with breast cancer: correlation with clinical data. *Cancer*, **48**, 876.

FORD, L.C., BEREK, J.S., LAGASSE, L.D. & 5 others. Estrogen and progesterone receptors in ovarian neoplasms. *Gynecol. Oncol.*, **15**, 299.

HAMERLYNCK, J. VTH, VERMORKEN, J.B. & VAN DER BURG M.E.L. (1985). Phase II study of tamoxifen in advanced ovarian cancer. *Proc. 3rd ECCO*, 43.

HAMILTON, T.C., YOUNG, R.C., MCKOY, W.M. & 7 others (1983). Characterisation of a human ovarian carcinoma cell line (NIH: OVCAR-3) with androgen and estrogen receptors. *Cancer Res.*, **43**, 5379.

HAWKINS, R.A., BLACK, R., STEELE, R.J.C., DIXON, J.M.J. & FORREST, A.P.M. (1981). Oestrogen receptor concentration in primary breast cancer and axillary node metastases. *Breast Cancer Res. Treat.*, **1**, 245.

HAWKINS, R.A., HILL, A. & FREEDMAN, B. (1975). A simple method for the determination of oestrogen receptor concentrations in breast tumours and other tissues. *Clin. Chimica Acta*, **64**, 203.

JONES, L.A., EDWARDS, C.L., FREEDMAN, R.S., TAN, M.T. & GALLAGHER, H.S. (1983). Estrogen and progesterone receptor titers in primary epithelial ovarian carcinomas. *Int. J. Cancer*, **32**, 567.

LANDONI, F., GHERLARDONI, C., ZANINI, A. & COLOMBO, N. (1983). Tamoxifen in advanced epithelial ovarian cancer. *J. Steroid Biochem.*, **19**, 935.

LANGDON, S.P., LAWRIE, S.S., HAY, F.G. & 7 others (1988). Characterisation and properties of 9 human ovarian adenocarcinoma cell lines. *Cancer Res.*, **48**, 6166.

LAZO, J.S., SCHWARTZ, P.E., MACLUSKY, N.J., LABAREE, D.C. & EISENFELD, A.J. (1984). Antiproliferative actions of tamoxifen to human ovarian carcinomas *in vitro*. *Cancer Res.*, **44**, 2266.

OSBORNE, R.J., MALIK, S.T., SLEVIN, M.L. & 4 others (1988). Tamoxifen in refractory ovarian cancer: the use of a loading dose schedule. *Br. J. Cancer*, **57**, 115.

PAGEL, J., ROSE, C., THORPE, S. & HALD, I. (1983). Treatment of advanced ovarian carcinoma with tamoxifen. A phase II trial. *Proceedings of 2nd European Conference on Clinical Oncology, Cancer Nursing (Amsterdam).*

POLLOW, K., SCHMIDT-MATTHIESEN, A., HOFFMAN, G. & 4 others (1983). $^{3}$H-Estradiol and $^{3}$H-R5020 binding in cytosols of normal and neoplastic human ovarian tisssue. *Int. J. Cancer*, **31**, 603.

REDDEL, R.R., MURPHY, L.C., HALL, R.E. & SUTHERLAND, R.L. (1985). Differential sensitivity of human breast cancer cell lines to the growth inhibitory effects of tamoxifen. *Cancer Res.*, **45**, 1525.

REDDEL, R.R., MURPHY, L.C. & SUTHERLAND, R.L. (1984). Factors affecting the sensitivity of T-47D human breast cancer cells to tamoxifen. *Cancer Res.*, **44**, 2398.

RUNGE, H.M., NEUMANN, H.A., BAUKNECHT, T. & PFLEIDERER, A. (1986). Growth patterns and normal sensitivity of primary tumor, abdominal metastasis and ascitic fluid from human epithelial ovarian carcinomas in the tumor colony forming assay. *Eur. J. Cancer Clin. Oncol.*, **22**, 691.

RUNGE, H.M., TEUFEL, G., NEULEN, J., GEYER, H. & PFLEIDERER, A. (1986). *In vitro* responsiveness of ovarian epithelial carcinomas to endocrine therapy. *Cancer Chemother. Pharmacol.*, **16**, 58.

SCHWARTZ, P.E., KEATING, G., MACLUSKY, N., NAFTOLIN, N. & EISENFELD, A. (1982). Tamoxifen therapy for advanced ovarian cancer. *Obstet. Gynecol.*, **59**, 583.

SHIREY, D.R., GERHENSON, D.M. & KAVANAUGH, J.J. (1984). Tamoxifen therapy of epithelial ovarian carcinoma. *Proc. Amer. Soc. Clin. Oncol.*, **170**, 3.

SLEVIN, M.L., HARVEY, V.J., OSBORNE, R.J., SHEPHERD, J.H., WILLIAMS, C.J. & MEAD, G.M. (1986). A Phase II study of tamoxifen in ovarian cancer. *Eur. J. Cancer Clin. Oncol.*, **22**, 309.

SLOTMAM, B.J. & RAO, B.R. (1988). Ovarian Cancer (Review). *Anticancer Res.*, **8**, 417.

STANLEY, E.R., PALMER, R.E. & SOHN, U. (1977). Development of methods for the quantitative *in vitro* analysis of androgen-dependent and autonomous Shionogi Carcinoma 115 cells. *Cell*, **10**, 35.

SUTHERLAND, R.L., HALL, R.E. & TAYLOR, I.W. (1983). Cell proliferation kinetics of MCF-7 human mammary carcinoma cells in culture and effects on exponentially growing and plateau phase cells. *Cancer Res.*, **43**, 3998.